# COVID-19 pneumonia: Prediction of patient outcome by CT-based quantitative lung parenchyma analysis combined with laboratory parameters

**DOI:** 10.1371/journal.pone.0271787

**Published:** 2022-07-29

**Authors:** Thuy D. Do, Stephan Skornitzke, Uta Merle, Maximilian Kittel, Stefan Hofbaur, Claudius Melzig, Hans-Ulrich Kauczor, Mark O. Wielpütz, Oliver Weinheimer

**Affiliations:** 1 Clinic for Diagnostic and Interventional Radiology (DIR), University Hospital Heidelberg, Heidelberg, Germany; 2 Translational Lung Research Center Heidelberg (TLRC), German Center for Lung Research (DZL), Heidelberg, Germany; 3 Department of Internal Medicine IV (Gastroenterology and Infectious Disease), University Hospital Heidelberg, Heidelberg, Germany; 4 Institute for Clinical Chemistry, Medical Faculty Mannheim of Heidelberg University, Mannheim, Germany; 5 Clinic for Gastroenterology and Nephrology, Landshut Hospital, Landshut, Germany; 6 Department of Diagnostic and Interventional Radiology with Nuclear Medicine, Thoraxklinik, University Hospital Heidelberg, Heidelberg, Germany; Public Library of Science, UNITED STATES

## Abstract

**Objectives:**

To evaluate the prognostic value of fully automatic lung quantification based on spectral computed tomography (CT) and laboratory parameters for combined outcome prediction in COVID-19 pneumonia.

**Methods:**

CT images of 53 hospitalized COVID-19 patients including virtual monochromatic reconstructions at 40-140keV were analyzed using a fully automated software system. Quantitative CT (QCT) parameters including mean and percentiles of lung density, fibrosis index (FIBI_-700_, defined as the percentage of segmented lung voxels ≥-700 HU), quantification of ground-glass opacities and well-aerated lung areas were analyzed. QCT parameters were correlated to laboratory and patient outcome parameters (hospitalization, days on intensive care unit, invasive and non-invasive ventilation).

**Results:**

Best correlations were found for laboratory parameters LDH (r = 0.54), CRP (r = 0.49), Procalcitonin (r = 0.37) and partial pressure of oxygen (r = 0.35) with the QCT parameter 75^th^ percentile of lung density. LDH, Procalcitonin, 75^th^ percentile of lung density and FIBI-_700_ were the strongest independent predictors of patients’ outcome in terms of days of invasive ventilation. The combination of LDH and Procalcitonin with either 75^th^ percentile of lung density or FIBI_-700_ achieved a r^2^ of 0.84 and 1.0 as well as an area under the receiver operating characteristic curve (AUC) of 0.99 and 1.0 for the prediction of the need of invasive ventilation.

**Conclusions:**

QCT parameters in combination with laboratory parameters could deliver a feasible prognostic tool for the prediction of invasive ventilation in patients with COVID-19 pneumonia.

## Introduction

Coronavirus disease 2019 (COVID-19) was declared a pandemic by the World Health Organization on March 11^th^ 2020 and has caused 27,339,132 infections and 892,648 deaths worldwide (as of September 8^th^ 2020) according to the Johns Hopkins dashboard [1]. The lung is the predominant organ affected by the disease presenting as pneumonia with rapid progression to severe respiratory distress syndrome requiring intensive care unit admission in up to 32% of cases [2, 3]. CT plays an essential role in early detection of pneumonia and can identify typical radiological patterns found in COVID-19: from ground-glass opacities in early stages to consolidation with a predominant peripheral distribution up to two weeks after disease onset [4, 5]. Sensitivities of chest CT for identification of COVID-19 of up to 98% have been reported and were shown to be superior to real-time polymerase chain reaction (RT-PCR) with sensitivities of 71% [6]. However, CT appearance is considered to be non-specific, though Bai et al. reported CT-specificity of 93% for distinguishing COVID-19 from other viral pneumonias [7]. Good correlations of CT features with severity of the disease and clinical parameters have been shown, e.g. 23–50% lung involvement with critical illness requiring ICU care [8–10].

Fully automatic quantification of structural lung disease, i.e. quantitative CT (QCT), has already been used as a feasible method for the evaluation of emphysema, airways disease, interstitial lung disease or atelectasis in large study population [11–17]. Many studies have shown laboratory parameters correlate with the degree of lung involvement in COVID-19 pneumonia using semi-quantitative or quantitative scoring, especially inflammation parameters such as C-reactive protein, D-dimer, interleukin-6, blood count or blood gas analysis [10, 18, 19].

We hypothesize that lung density changes such as ground-glass opacities and consolidation in COVID-19 pneumonia can be captured by QCT and that the extent of these changes may be not only related to disease severity, but also allows for prognostication in combination with clinical and laboratory parameters at the time of hospital admission.

Therefore, the aim of this study was to quantify early and advanced signs of COVID-19 pneumonia by attenuation-based QCT and correlate QCT parameters as well as established laboratory parameters with patient outcome.

## Material and methods

### Study design and patient recruitment

This retrospective exploratory single center study was performed according to the Declaration of Helsinki. Ethical approval was obtained from the local ethical review board of the Medical Faculty University Hospital Heidelberg with approval number S-293/2020. The need for written informed consent was waived.

### Clinical data selection and study design

Eighty-six patients were referred to our institution with symptoms of COVID-19 pneumonia and admitted to the isolation ward between March and May 2020 were retrieved from the radiological information system (Centricity RIS, GE Healthcare) and eligible for study inclusion. Inclusion criteria were one positive RT-PCR test and at least one chest CT with abnormal findings. 17 patients with other causes for pneumonia, such as influenza A and B proven by RT-PCR, were excluded. 15 patients were excluded due to contrast media application. One patient with streaking artifacts due to extensive breathing artifacts was excluded. To ensure comparability and homogeneity of acquisition settings, only 53 patients examined with the same CT-scanner and non-contrast enhanced acquisition protocol were included into the study.

Patients’ clinical information and laboratory parameters were obtained from the hospital information system (I.S.-H.*med., SAP). Clinical information included date of CT, symptoms, date of symptom onset, admission and duration of hospitalization/intermediate care/intensive care unit, need and duration of invasive and non-invasive ventilation. Laboratory parameters within 24 hours of the CT included creatine kinase (CK), leucocytes, thrombocytes, neutrophile granulocytes, eosinophil granulocytes, lymphocytes, lactate dehydrogenase (LDH), C-reactive protein (CRP), interleukine-6 (IL-6), Procalcitonin and coagulation parameters. Blood gas analysis parameters i.e. partial pressure of oxygen (PaO2), partial pressure of carbon dioxide (PaCO2), pH-value, base deficit/excess and lactate were also retrieved. Only blood gas analysis that was closest to the time point of CT imaging with a maximum of 24 hours time interval was taken into account.

### CT protocol, reconstruction and image postprocessing

Non-contrast enhanced chest CT was performed on a dual-layer detector spectral CT scanner (IQon, Philips). Patients were in supine position and acquisitions were obtained at end-inspiratory breath-hold achieving near total lung capacity and preventing atelectasis. Acquisition parameters were as follows: collimation 64 x 0.625 mm, tube potential 120 kV_p_, automated tube current modulation, reference tube current 47 mAs, pitch 1.0, dose right index 9 and CTDI_vol_ 3.8 mGy. Axial reconstructions with 1.5 mm slice thickness und 0.75 mm interval with lung kernel (YB) and soft tissue kernel (IMR1) were performed. In addition, pseudo-monoenergetic images from 40 to 140 keV in steps of 20 keV and reconstruction kernel B were generated using manufacturer’s dedicated image postprocessing software (IntelliSpace, Philips).

### Quantitative image analysis

Quantitative image analysis was performed with YACTA (version 2.9.0.31), a non-commercial, well-evaluated scientific software as previously described [11, 16, 17, 20–23]. YACTA segmented and analyzed the airway tree, the vessels and the lungs fully automatically. Ye, et al. described chest CT manifestation of COVID-19 in line with terms defined by the Fleischner Society such as ground-glass opacity, consolidation and crazy paving pattern [4]. We chose the following QCT parameters as COVID-19 markers:

1. Mean lung density (MLD), defined as the average CT values of all segmented lung voxels. The CT values are closely related to the lung density [24].
2. i^th^ percentile of the lung histogram, defined as the CT value such that i percent of the lung voxels are less than or equal to that CT value. In [22] is shown that percentiles might reflect changes in lung abnormalities.
3. Fibrosis index (FIBI_-700_), defined as the percentage of the segmented lung voxels ≥ -700 HU, voxels labeled as vessel are excluded. This index is intended to describe the proportion of consolidations.
4. Ground-glass opacity index (GGOI_-800_), defined as the percentage of the segmented lung voxels ≥−800 HU and <-700 HU. This index is intended to describe the proportion of GGO regions that can be seen as a precursor to consolidations.
5. Healthy lung index (HLI_-700_), defined as the percentage of the segmented lung voxels ≥-950 HU and <-700 HU [18].
6. Wall%, defined as average of the quotient airway wall area and the total airway area for the whole segmented airway tree [20].
7. AWT-Pi10, defined as the square root of the airway wall area for a ‘theoretical airway’ with an internal perimeter of 10 mm [25].
8. Central and peripheral vessel volume. Therefore, the lung was divided into inner (core) and outer (in the rind) region, central vessels are located in the core region, peripheral vessels int the rind region [26].

Note, that the chosen GGOI_-800_ interval is part of the HLI_-700_ interval, to identify whether this partial HU interval can provide additional information on an incipient COVID-19 disease. The image analysis was performed for the reconstructions with soft tissue kernel (IMR1), lung kernel(YB) and all six pseudo-monochromatic reconstructions (VMSI) from 40 to 140 keV (B).

### Statistical analysis

Statistical analyses were performed using RStudio (R version 4.0.2). Pearson’s correlation for linear correlation between QCT parameters of all different lung reconstructions (IMR1, YB, VMSI 40–140 keV B), laboratory values and interval scaled clinical outcome parameters were computed. For correlation of binary variables (invasive and non-invasive ventilation) with metric variables from QCT and laboratory parameters the pseudo r^2^ (McFadden’s r^2^) was calculated. Multiple linear regression and logistic regression with backward, forward and bidirectional elimination was performed to achieve the best combination of laboratory, QCT and clinical parameters to predict different clinical endpoints. The Akaike Information Criterion (AIC) was used to identify the best model [27]. Furthermore, receiver operating curve (ROC) analysis was done and the area under the ROC curve (AUC) was calculated to determine the model performance of logistic regression analysis. The significance level for statistical testing was set at p<0.05, because of the exploratory nature of the study the Benjamini-Hochberg method was used for adjustment for multiple testing [28].

## Results

### Patient population

The mean age of the study population was 59.9 ±14 years and the patient collective comprised 19 females and 34 males (**Table 1**). Average time between onset of symptoms and initial CT imaging was 7.1 ± 4.9 days (median: 7.0 days). CT imaging was routinely performed on the first day of admission. Main initial symptoms were fever (88%), cough (81%), dyspnea (50%), diarrhea (35%), pain in the limbs (26%) and fatigue (24%). Among the 53 hospitalized patients with a CT scan five patients were admitted to an isolation ward and 48 patients on intermediate or intensive care unit. On average patients were hospitalized for 12.2 ± 6.8 days (median: 12.0 days) and spent 10.2 ±6.4 days (median: 8.0 days) on intermediate care or intensive care unit. Those who needed respiratory support were ventilated non-invasively for a mean of 5.3 ± 3.7 days (median: 4 days; 15 patients) or invasively for 14.6 ±9.6 days (median: 14 days; 11 patients), respectively.

**Table 1:** Characteristics of study population.

Characteristics of study population (n = 53)	Mean and SD
**Age [years]**	59.9 ± 14.0
**Sex [n (%)]**	
**Female**	19 (36%)
**Male**	34 (64%)
**Length of hospitalization [days]**	12.2 ± 6.8
**Length patient care on ICU/ICM [days]**	10.2 ± 6.4
**Need for non-invasive ventilation [n (%)]**	14 (26.4%)
**Length of non-invasive ventilation [days]**	5.3 ± 3.7
**Need for invasive ventilation [n (%)]**	11 (20.6%)
**Length of invasive ventilation [days]**	14.6 ± 9.6

### Laboratory testing

Laboratory studies with mean values and standard deviation (**Table 2**) demonstrated lymphopenia 0.83/nl (normal range: 1.0–4.8/nl); D-dimer was elevated 1.19 mg/dl (normal range <0.5 mg/dl); Additional parameters included thrombocytes, leucocytes and coagulation parameters (pTT, INR) were within the normal range. There was an increase of CRP 81.6 mg/l (normal range <5 mg/l) and LDH 391.28 (normal range <317 U/l). Pathologic blood gas analysis was noticeable in 14 cases (26%), from which in 11 cases were respiratory alkalosis and three cases of respiratory azidosis.

**Table 2:** Mean and standard deviation of the laboratory parameters of all 53 COVID-19 patients.

Laboratory parameter	Mean and SD of 53 patients	Normal range
Leucocytes	6.17 ± 3.02/nl	4-10/nl
**Lymphocytes**	0.83 ± 0.36/nl	1.0–4.8/nl
**Neutrophilic granulocytes**	4.81 ± 2.91/nl	1.8–7.7/nl
**Eosinophilic granulocytes**	0.44 ± 0.08/nl	<0.5/nl
**Thrombocytes**	209.38 ± 76.45/nl	150-440/nl
**Lactate dehdydrogenase**	391.28 ± 128.94 U/l	<317 U/l
**C-reactive protein**	81.65 ± 82.05 mg/l	<5 mg/l
**Interleukine-6**	473.46 ± 296 pg/ml	<15 pg/ml
**Procalcitonin**	0.37 ± 1.30 ng/ml	<0.05 ng/ml
**International Normalized Ratio**	1.10 ± 0.25	<1.2
**Prothrombin time**	25.15 ± 3.56 s	<35 s
**D-Dimer**	1.19 ± 1.83 mg/l	<0.5 mg/l
**Creatine kinase**	168.47 ± 213.73 U/l	<190 U/l

### QCT analysis

**Fig 1:**
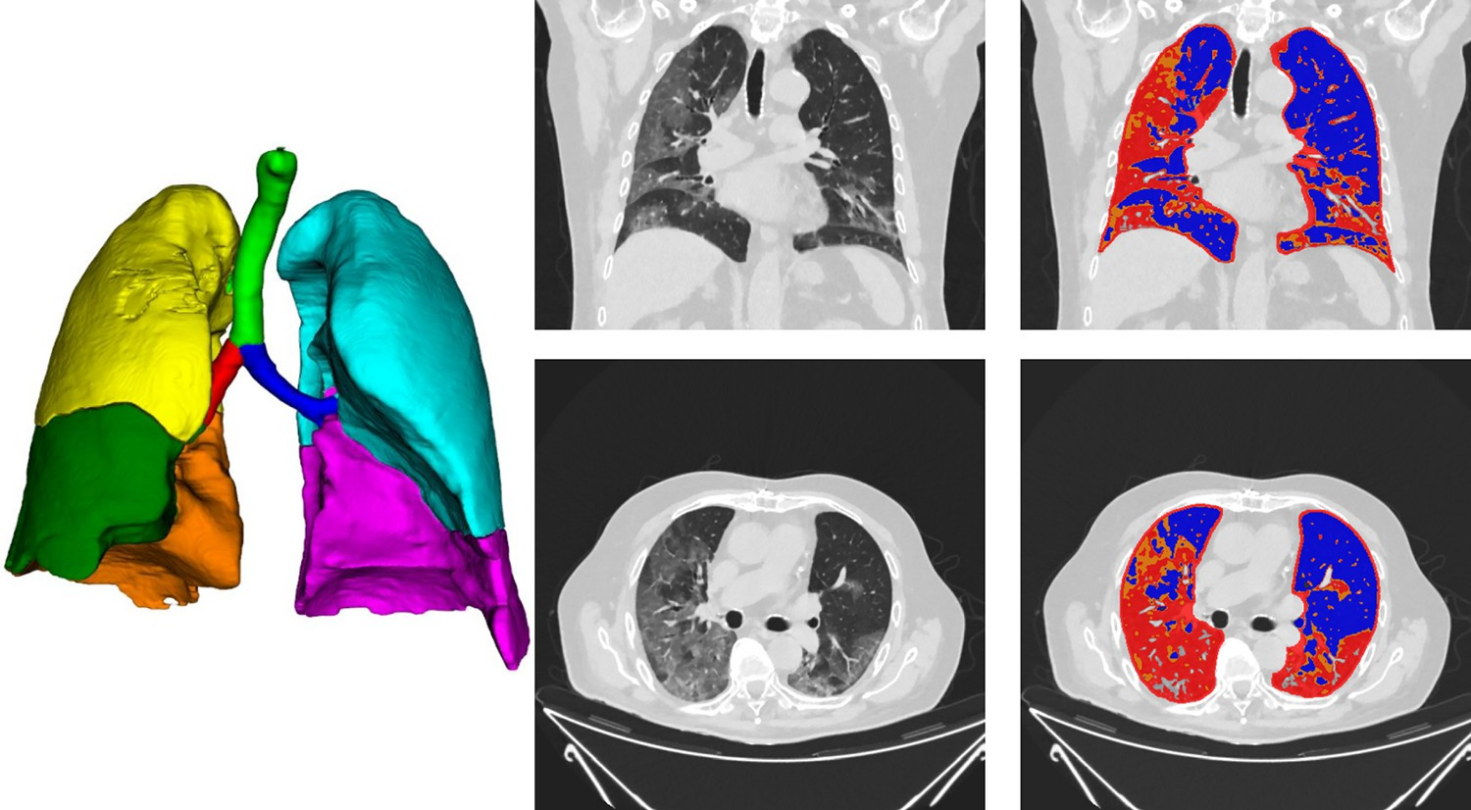
The YACTA software performed a fully automatic segmentation of airways, vessels and lung parenchyma of an 84y/o male with extensive lung infiltrates who received high-flow oxygen therapy and invasive ventilation. In the course of the disease he developed renal insufficiency, septic shock and died 21 days after symptom onset and 8 days after hospital admission. Consolidations are shown in red, ground-glass opacities in orange, healthy lung in blue, vessel voxels in grey are excluded. The 3D rendering shows the lung segmentation with their segmented lobes in different colors, in the statistical evaluation the lung parenchyma was considered as a whole.

All QCT parameters are shown in **Table 3** and an example of automatic lung segmentation without manual correction is presented in **Fig 1**. We found only slight differences in the QCT parameters for the different reconstructions. In the following, we therefore focus only on the QCT results generated for IMR1 reconstructions, as we saw the best correlation between QCT and laboratory parameters there.

**Table 3:** QCT parameters of 53 COVID-19 patients in the soft kernel images IMR1.

QCT parameters	Mean and SD of 53 patients
**Mean lung density**	-681.19 ± 78.30 HU
**80th percentile lung density**	-544.26 ± 167.59 HU
**75th percentile lung density**	-621.47 ± 158.52 HU
**70th percentile lung density**	-674.75 ± 144.18 HU
**FIBI_-700_**	30.55 ± 14.47%
**GGO_-800_**	16.35 ± 9.70%
**HLI_-700_**	66.10 ± 15.01%
**Wall%**	52.59 ± 5.34%
**AWT-Pi10**	0.31 ± 0.13 mm
**Central vessel volume**	129.31 ± 67.12 cm^3^
**Peripheral vessel volume**	31.23 ± 27.28 cm^3^

The mean lung density was -681.2 ±78.3 HU, the 75^th^ percentile lung density -621.5 ±158.5 HU, the mean of FIBI_-700_ was 30.6 ±14.47%, GGOI_-800_ was 16.4 ±9.7% and HLI_-700_ 66.1 ±15.0%. Percentage of airwall thickness was 0.31 ±5.34% and AWT-Pi-10 0.31 ± 0.13 mm. Central and peripheral vessel volume were 129.31 ± 67.12 cm^3^ and 31.23 ± 27.28 cm^3^, respectively.

### Correlation analysis

**Fig 2:**
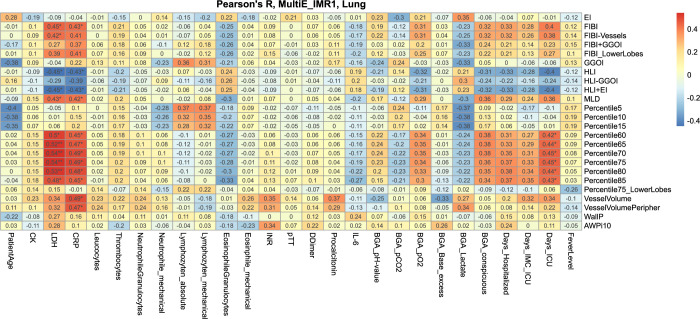
Heat map of Pearson’s correlation between QCT lung segmentation (y-axis) and laboratory parameters and clinical outcome (x-axis). Significant codes: *** p < 0.001 / ** p < 0.01 / * p < 0.05.

**Fig 3:**
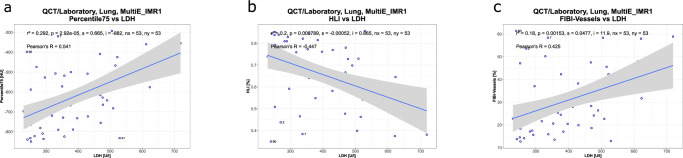
Correlations between LDH and QCT parameters with highest Pearson‘s r for 75^th^ lung percentile in comparison to HLI_-700_ and FIBI_-700_.

Next, we correlated QCT parameters with laboratory work, blood gas analysis, body temperature, length of hospitalization and intermediate/intensive care unit and, length of invasive and non-invasive ventilation (**Fig 2 and S1 Appendix**). Comparing QCT parameters, the strongest correlation with laboratory and clinical parameters was observed for 70-80^th^ lung percentiles (Pearson’s r up to 0.54; p<0.01), HLI_-700_ (r = -0.45; p<0.05), mean lung density (r = 0.43; p<0.05), FIBI_-700_ (r = 0.45; p<0.05) and central and peripheral vessel volume (r = 0.49 and 0.47, respectively; p<0.05) (**Figs 2 and 3**). Other QCT parameters e.g. GGOI_-800_, Wall% and AWT-Pi10 showed lower or inversed correlations. Central vessel volume and peripheral vessel volume correlated strong positively with CRP (r = 0.49–0.47; p<0.05).

Strongest correlation between QCT parameters and laboratory data was observed for the soft tissue kernel IMR1 and slightly lower correlations were observed for the lung kernel YB (S2 Appendix) and virtual monoenergetic images 40–140 keV (S3-S8 Appendices).

Among laboratory parameters LDH (r = 0.54; p<0.01), CRP (r = 0.49; p<0.05), Procalcitonin (r = 0.37; p>0.05), and partial pressure of oxygen (r = 0.35; p>0.05) demonstrated the highest correlation coefficients with quantitative lung segmentation parameters such as 70^th^ or 75^th^ lung percentiles.

**Fig 4:**
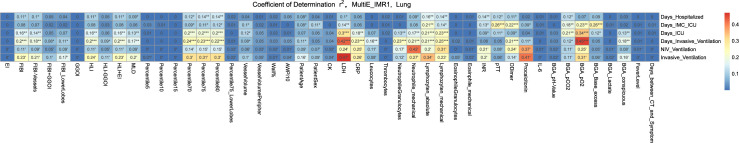
Heat map of coefficient of determination r^2^ for the correlation of outcome parameters with laboratory and QCT parameters. For binary outcome parameters (NIV_Ventilation and Invasive_Ventilation) the Mc Fadden pseudo r^2^ was calculated, there is no p-value to specify for this value. Signif. codes: *** p < 0.001 / ** p < 0.01 / * p < 0.05 / ’ no p-value available.

Pseudo r^2^ was calculated for correlation of binary outcome parameters with laboratory and QCT parameters (**Fig 4**). Highest correlation of McFadden pseudo r^2^ for whether patients were invasively or non-invasively ventilated was 0.47 for LDH and 0.41 for Procalcitonin. Length of hospitalization correlated with QCT percentiles of lung density (r^2^ = 0.14, p<0.01). Length of invasive ventilation, non-invasive ventilation and days on intensive and intermediate care unit showed the highest correlation with partial oxygen pressure (r^2^ = 0.45; p<0.001) and LDH (McFadden pseudo r^2^ = 0.47) when evaluating laboratory parameters and with 70-80^th^ percentile lung density (McFadden pseudo r^2^ = 0.31) when evaluating quantitative lung segmentation parameters.

The backward elimination technique led to the best multiple linear regression model for the prediction of days of invasive ventilation according to the Akaike information criterion (AIC) leading to an adjusted r^2^ of 0.72 with a p-value of 8.63e-10, detailed results see in S9 Appendix.

### Combined prediction model

**Fig 5:**
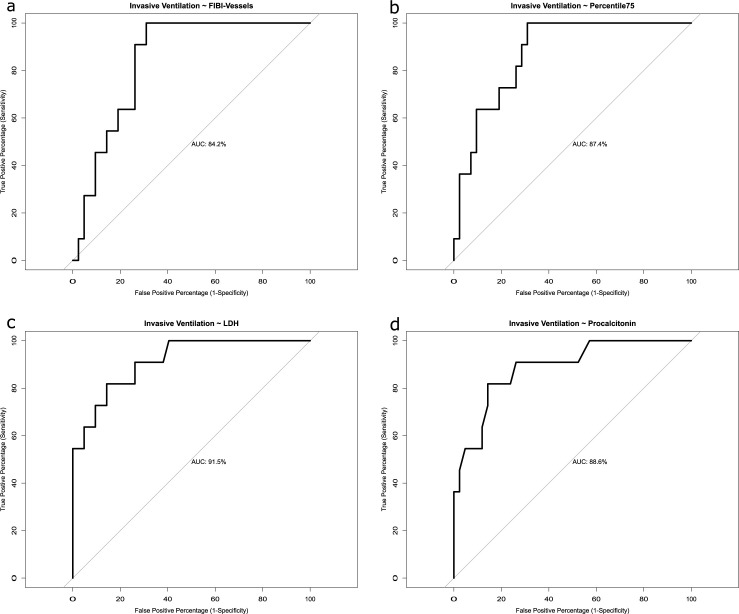
ROC curves of invasive ventilation QCT parameters alone with FIBI_-700_ (a) vs. 75^th^ percentile lung density (b) vs. LDH (c) vs. Procalcitonin (d).

The predictive value of QCT and laboratory parameters for the necessity of invasive ventilation was investigated. ROC analysis was applied and the AUC was calculated to determine model performance. AUC value for ROC analysis based on simple logistic regression was 84.2% for FIBI_-700_ (McFadden pseudo r^2^ = 0.21) and the best threshold delivered a true positive percentage (TPP, sensitivity) of 100% and a false positive percentage (FPP, 1-specificity) of 31%. 75^th^ lung percentile parameter summed up to an AUC of 87.4% (r^2^ = 0.31, TPP = 100%, FPP = 31%). AUC values for LDH were 91.5% (r^2^ = 0.47, TPP = 82%, FPP = 14%) and for Procalcitonin 88.6% (r^2^ = 0.37, TPP = 82%, FPP = 14%) for (**Fig 5**).

**Fig 6:**
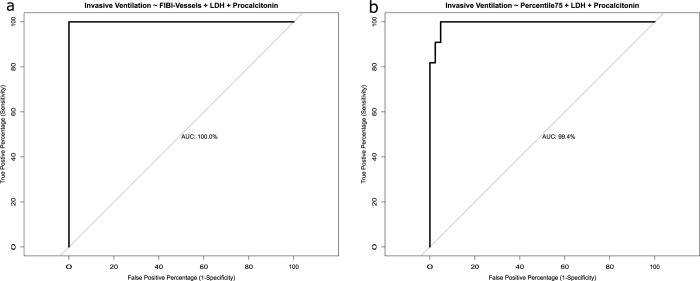
ROC curves for invasive ventilation consisting of the combination of QCT and laboratory work: FIBI_-700_, LDH and Procalcitonin; (b) 75^th^ percentile lung density, LDH and Procalcitonin.

Backward elimination technique was used to identify the best multiple logistic regression model. The combination of FIBI_-700_ with LDH and Procalcitonin achieved an AUC of 100% (r^2^ = 1, TPP = 100%, FPP = 0%). The combination of 75^th^ percentile lung density with LDH and Procalcitonin yielded an AUC of 99.4% (r^2^ = 0.84, TPP = 100%, FPP = 5%, **Fig 6**).

## Discussion

The aim of the study was to correlate the outcome of hospitalized patients with COVID-19 pneumonia with initial laboratory and imaging findings to identify predictive markers for disease progress. Correlations of initial QCT parameters of COVID-19 pneumonia with clinical parameters, such as days on intensive and/or intermediate care unit, and laboratory parameters, e.g. LDH, CRP, Procalcitonin and blood gas analysis, were observed. Thus, initial CT and laboratory testing could aid to stratify patient treatment and indicate patients might require closer monitoring at the time of hospital admission. Taken together, both could help to find solutions to provide cancer patients with better care [29, 30]. We showed that laboratory parameters Procalcitonin and LDH as well as QCT parameters FIBI_-700_ and 75^th^ percentile of lung density are predictors for the need for invasive ventilation. The combination of QCT parameters and laboratory work shows superior results for the prediction of the necessity of invasive ventilation. Though blood gas analysis and many other laboratory parameters were taken into account for establishing a prediction score, only Procalcitonin and LDH showed significant results for establishing a prognostic score. Either a combination calculated from 75^th^ percentile of lung density, Procalcitonin and LDH or from FIBI_-700_, Procalcitonin and LDH could be used to estimate the need for invasive ventilation.

Contrary to expectations, D-Dimer was only slightly elevated and did not show high correlations to any morphological lung segmentation parameters. This might be explained by the fact that in early stages of the diseases D-Dimer elevation is associated with inflammation and prothrombotic state and embolism are seen in ICU patients or patients with critical illness only when D-Dimer is markedly elevated [31]. Moreover, preventive anticoagulation is not recommended for COVID-19 for outpatients and low molecular weight heparin or unfractionated heparin may be given in hospitalized patients with severe illness [32, 33]. Another factor might be using non-contrast chest scans which could exclude patients with a severe disease manifestation.

Colombi et al. proposed the usage of well-aerated lung as a parameter to predict outcome. Unfortunately, no additional QCT parameters were examined and thus potential tools for prognostication may be missed [34]. However, the method used in their study still required manual adjustments, which might limit its upscaling to a larger study population. Moreover, in this study the approach included the 75^th^ percentile of lung density, FIBI-_700_ and normally-aerated lung proportion which showed comparable correlations with clinical parameters and outcome parameters. However, only FIBI_-700_ in combination with LDH and Procalcitonin achieved an AUC of 100%. Wall% and AWT-Pi10 did not correlate well with laboratory parameters, as the pulmonary manifestations focuses on the lung parenchyma. In line with previous studies dilated vessel volumes on non-enhanced CT images has been reported and might be explained by small pulmonary embolism or paradoxical increase of blood flow [35, 36].

Several approaches have been made for automated or artificial intelligence-based lung segmentation in COVID-19 diseases to predict adverse outcome,ARDS only or correlation of lung segmentation with laboratory works, but did not use a multifactorial approach [22, 34, 37–39]. Park et al. have performed the first multifactorial approach to evaluate prognostic implication of lung segmentation and laboratory work, especially CRP, on outcome parameters [40]. However, manual adjustment was performed for the lung segmentation using commercially available software such as modifying parenchymal segmentation and exclusion of parenchymal lesions (honeycombing, bronchiectasis, pleural effusion and others). Our approach offers a fully-automated segmentation with an openly available algorithm without any further manual adjustments, as the threshold chosen already excludes those parenchymal lesions as mentioned above. The method has been used for different CT devices in previous studies and offers the opportunity for upscaling to a lager or nationwide population.

Gattinoni et al. suggest to adapt the ventilation according to the lung weight by differentiating them in low and high lung weight [41]. Fully automated segmentation of the lung might facilitate the decision for the mode of ventilation and should be the subject of further investigations.

CT images reconstructed with the soft tissue kernel showed better correlations with laboratory and clinical parameters than the lung kernel or VMSI, which is in line with recommendations for lung segmentation of previous studies [42, 43]. All six VMSI reconstructions and lung kernel were slightly inferior to the soft tissue kernel in CT without contrast agent in this study. However, VMSI might be useful combined with contrast agent in patients with severe symptoms in advanced disease stage, especially with COVID-19 to detect associated thromboembolic complications [44, 45].

The most important limitation of the study was the modest number of patients and the retrospective nature of the study. Our results require further verification in a prospective trial. However, only patients hospitalized with a positive SARS-COV2 test confirmed by PCR were included and all patients received the same CT acquisition protocols at the same CT device. In general, the low number of included patients might be attributed to the overall low infection numbers in Germany, especially in this geographical region, at the time of the study. The segmentation algorithm has been employed for previous studies with different CT devices. Based on previous results, we estimate that the fully automatic segmentation can be transferred to a large multicentric cohort on a nationwide level with acceptable effort, provided that a national platform for images and clinical data is established. A wide range of laboratory parameters and gas blood analysis were included into the correlation analysis and prediction score establishment, and laboratory work was not standardized with sporadically missing values.

## Conclusion

An independent correlation with clinical outcome parameters for COVID-19 could be shown for a wide range of laboratory parameters, with strongest correlations for CRP, Procalcitonin and LDH.

QCT parameters 70^th^-80^th^ percentile of lung density and FIBI_-700_ correlated best with clinical parameters and outcome. Fully QCT and laboratory testing are independently predictive factor for invasive ventilation. However, Procalcitonin and LDH in combination either with the 75^th^ percentile of lung density or FIBI_-700_ achieved highest prognostic value for invasive ventilation derived from initial CT imaging.

## Supporting information

S1 Appendix Heat map of all QCT lung segmentation, laboratory and clinical outcome parameters. (EPS)

S2 Appendix Heat map of Pearson‘s correlation for lung kernel. (EPS)

S3 Appendix Heat map of Pearson‘s correlation for virtual monochromatic reconstruction at 140 keV. (EPS)

S4 Appendix Heat map of Pearson‘s correlation for virtual monochromatic reconstruction at 120 keV. (EPS)

S5 Appendix Heat map of Pearson‘s correlation for virtual monochromatic reconstruction at 100 keV. (EPS)

S6 Appendix Heat map of Pearson‘s correlation for virtual monochromatic reconstruction at 80 keV. (EPS)

S7 Appendix Heat map of Pearson‘s correlation for virtual monochromatic reconstruction at 60 keV. (EPS)

S8 Appendix Heat map of Pearson‘s correlation for virtual monochromatic reconstruction at 40 keV. (EPS)

S9 Appendix Result of best multiple linear regression model of QCT and laboratory parameter with clinical outcome parameters. (DOCX)

## Abbreviations

BGA: Blood gas analysis
COVID-19: Coronavirus disease 2019
CK: Creatine kinase
CRP: C-reactive protein
CT: Computed tomography
HU: Hounsfield Unit
ICU: Intensive care unit
IL-6: Interleukine-6
IMC: Intermediate care
LDH: Lactate dehydrogenase
RT-PCR: Real-time reverse transcription polymerase chain reaction
SARS-CoV-2: Severe acute respiratory syndrome coronavirus 2
