# Supplementary material for: COVID-19 pneumonia: Prediction of patient outcome by CT-based quantitative lung parenchyma analysis combined with laboratory parameters
Source: PLoS One. 2022 Jul 29;17(7):e0271787. doi: 10.1371/journal.pone.0271787 (PMC9337660; doi:10.1371/journal.pone.0271787)
Supplement: S9 Appendix — (DOCX) [file pone.0271787.s009.docx]

Result of best multiple linear regression model (with R 4.0.2)

beta(Final) #Best model

Residuals:

Min 1Q Median 3Q Max

-1.03444 -0.26502 0.00011 0.31728 1.12703

Coefficients:

Estimate Std. Error t value Pr(>|t|)

(Intercept) 1.799e-16 7.288e-02 0.000 1.00000

FIBI.z 5.882e-01 3.140e-01 1.874 0.06832 .

Percentile75.z -4.325e-01 3.346e-01 -1.293 0.20351

LDH.z 3.679e-01 1.036e-01 3.550 0.00100 **

NeutrophileGranulozyten.z 4.682e-01 1.441e-01 3.249 0.00235 **

Lymphozyten_mechanisch.z 2.439e-01 1.523e-01 1.602 0.11711

Lymphozyten_absolut.z -3.346e-01 1.262e-01 -2.652 0.01141 *

INR.z 1.872e-01 9.499e-02 1.971 0.05573 .

pTT.z 1.195e-01 9.212e-02 1.297 0.20216

D_Dimer.z 2.317e-01 8.870e-02 2.612 0.01261 *

Procalcitonin.z -2.849e-01 1.348e-01 -2.113 0.04092 *

BGA_pO2.z 1.461e-01 1.093e-01 1.337 0.18869

BGA_Base_excess.z -1.977e-01 8.215e-02 -2.406 0.02084 *

---

Signif. codes: 0 ‘***’ 0.001 ‘**’ 0.01 ‘*’ 0.05 ‘.’ 0.1 ‘ ’ 1

Residual standard error: 0.5306 on 40 degrees of freedom

Multiple R-squared: 0.7834, Adjusted R-squared: 0.7185

F-statistic: 12.06 on 12 and 40 DF, p-value: 8.63e-10
